# Characteristics and clinical courses of patients with atypical haemolytic uraemic syndrome on dialysis withdrawal after eculizumab treatment: sub-analysis of post-marketing surveillance in Japan

**DOI:** 10.1007/s40620-025-02433-z

**Published:** 2025-10-28

**Authors:** Shuichi Ito, Masanori Matsumoto, Akihiko Shimono, Hirofumi Teranishi, Shoichi Maruyama

**Affiliations:** 1 Department of Pediatrics, Graduate School of Medicine, Yokohama City University, Kanagawa, Japan; 2 Department of Hematology and Blood Transfusion Medicine, Nara Medical University, Nara, Japan; 3 Medical Affairs Division, Alexion Pharma GK, Tokyo, Japan; 4 Department of Nephrology, Nagoya University Graduate School of Medicine, Aichi, Japan

**Keywords:** Dialysis, Atypical haemolytic uraemic syndrome, Eculizumab, Post-marketing surveillance

## Abstract

**Background:**

Atypical haemolytic uraemic syndrome (aHUS) leads to acute kidney injury, necessitating dialysis in about half of patients. A certain proportion of patients treated with C5 inhibitors discontinue dialysis; however, little is known about the patient characteristics and clinical courses relating to discontinuation.

**Methods:**

We compared the characteristics and clinical courses of patients with aHUS on dialysis at the initiation of eculizumab during post-marketing surveillance in Japan, stratified by those who did (Group A) and did not (Group B) discontinue dialysis within 26 weeks of eculizumab treatment.

**Results:**

Of 38 included patients, 21 (55.3%) and 17 (44.7%) were placed in Groups A and B, respectively. No patient re-started dialysis. Hypertension was less frequent in Group A than in Group B (6/21 [28.6%] vs. 11/17 [64.7%], *p* = 0.022). Both the duration of dialysis before eculizumab initiation (6 vs. 17 days, *p* = 0.011) and the time from thrombotic microangiopathy onset to eculizumab initiation (9 vs. 25 days, *p* = 0.008) were shorter in Group A. A duration of less than 15 days from thrombotic microangiopathy onset to eculizumab initiation was associated with dialysis discontinuation. Kidney function improvement and normalisation of platelet count and lactate dehydrogenase levels were achieved earlier in Group A than in Group B (*p* = 0.050, 0.014, and < 0.001, respectively). Five (29.4%) of 17 patients in Group B discontinued dialysis after 27 weeks of eculizumab treatment, including one patient who underwent kidney transplantation.

**Conclusions:**

Early initiation of eculizumab was significantly correlated with dialysis discontinuation.

**Graphical Abstract:**

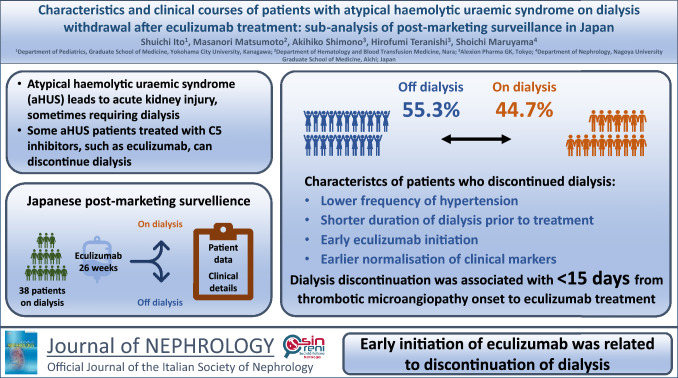

**Supplementary Information:**

The online version contains supplementary material available at 10.1007/s40620-025-02433-z.

## Introduction

Thrombotic microangiopathy (TMA) is characterised by microangiopathic haemolytic anaemia, consumptive thrombocytopenia, and organ damage, typically acute kidney injury (AKI). Atypical haemolytic uraemic syndrome (aHUS) is a TMA associated with dysregulation of the complement pathway [1, 2]. In aHUS, acute renal exacerbation leads to dialysis in approximately 50% of patients [3, 4]. High serum creatinine level, high mean arterial pressure, and mildly decreased platelet count at baseline have been identified as predictive characteristics for poor renal prognosis, including kidney failure, in patients with aHUS [5].

Currently, two drugs, eculizumab and ravulizumab, are approved for aHUS treatment in Japan; both are C5 inhibitors that bind to complement C5 and block cleavage to C5a and C5b, thereby preventing the formation of the membrane attack complex [6–8]. Previous studies have reported the recovery of kidney function in patients with aHUS after C5 inhibitor treatment [8–15]. These studies also reported that 50–80% of patients on dialysis at baseline discontinued dialysis after treatment with C5 inhibitors [6–11, 14].

Diagnosis of aHUS is complex, which may cause delays in initiation of appropriate treatment. In a post hoc analysis of an eculizumab clinical trial, early treatment initiation with eculizumab was related to an improvement in mean estimated glomerular filtration rate (eGFR) [15]. Real-world evidence studies also demonstrated that shorter time between disease presentation and the first dose of eculizumab was associated with improved kidney function [12, 16].

Eculizumab was approved for the treatment of aHUS in Japan in 2013. The post-marketing surveillance was commenced as a regulatory-mandated study to evaluate the real-world safety and effectiveness of eculizumab in Japan. Patients diagnosed with aHUS who received at least one dose of eculizumab were enrolled between September 2013 and July 2018. Data concerning patient characteristics and clinical course were collected, and the results confirmed the effectiveness and safety of eculizumab in a real-world setting [17–20]. In the current analysis, only patients who were on dialysis at the initiation of eculizumab treatment were included, and their characteristics and clinical courses were compared between patients who discontinued dialysis within 26 weeks of initiation of eculizumab and those who did not.

## Materials and methods

### Study design and treatment

This post-marketing surveillance is a regulatory-mandated study requested by the Ministry of Health, Labour and Welfare (MHLW) of Japan. Requirements of ethical approval and informed consent from patients were waived (MHLW Ministerial Ordinance No. 171 of 2004). The detailed methodology has previously been published [16–19]. Briefly, the post-marketing surveillance data collection period began in September 2013 and ended in July 2018, and the data were locked in August 2021. The post-marketing surveillance enrolled all patients clinically diagnosed with aHUS according to the latest clinical guide [20–22] and who had received at least one dose of eculizumab.

### Patients

The patients included in this study were paediatric and adult patients diagnosed with aHUS who were treated with eculizumab for ≥ 12 weeks. Patients with haematopoietic stem cell transplantation TMA, malignancy-related TMA, drug-induced TMA and autoimmune disease TMA were excluded because comorbidities might affect kidney function and dialysis continuation in a manner unrelated to aHUS. Patients whose treatment period was < 12 weeks were also excluded. The population included in this study was divided into two groups: patients who discontinued dialysis within 26 weeks from initiation of eculizumab (Group A) and patients who did not discontinue dialysis within that period (Group B).

### Data collection

The post-marketing surveillance collected information on patient characteristics, medical history, clinical courses before treatment and laboratory test values, and haematologic and renal parameters, at the initiation of eculizumab (baseline) and at 6 months, 12 months, and annually thereafter during eculizumab treatment. The patient characteristics analysed in the current study included age, sex, past medical and family history of aHUS, genetic variants, and the presence or absence of anti-complement factor H antibody. We assessed the percentage of patients with normalisation of both platelet count (platelet count ≥ 150 × 10^9^/L in ≥ 2 consecutive measurements over ≥ 4 weeks during the observation period) and lactate dehydrogenase (LDH) levels (below the upper limit of normal in ≥ 2 consecutive measurements over a period of at least 4 weeks during the observation period), and with renal improvement (decrease in serum creatinine from baseline of ≥ 25%, or change in eGFR from baseline of ≥ 15 mL/min/1.73 m^2^ in ≥ 2 consecutive measurements over 4 weeks during the observation period) over the treatment period.

### Statistical analysis

Data were analysed using median, quartiles (Q) 1–Q3 (for continuous variables), and frequency and proportions (for categorical variables). The baseline characteristics of patients were analysed using descriptive statistics and compared using the Wilcoxon’s rank sum test and Fisher’s exact test, as appropriate. Absolute values in platelet count, LDH levels, and eGFR were summarised using descriptive statistics, and changes from baseline were evaluated by the Wilcoxon signed-rank test. The percentages of patients with normalisation of platelet count, LDH levels, and renal improvement over the treatment period were estimated by Kaplan–Meier analysis. For the analysis of items associated with dialysis discontinuation, logistic regression analysis, using each analysis item as the response variable and the candidate covariates as explanatory variables, was performed using univariate and stepwise methods. Odds ratios (ORs), two-sided 95% confidence intervals (CIs) for ORs, and *p*-values were calculated for each item. In the multivariate analysis, the age at initiation of eculizumab, the presence or absence of hypertension as a complication, and the time from TMA onset to initiation of eculizumab treatment (< 15 days/ ≥ 15 days; median days from TMA onset to initiation of eculizumab treatment in the total population in this study) were included to examine the association with dialysis discontinuation. The significance level was set at 0.05, and the tests were two-sided. Statistical analyses were performed with SAS version 9.1.3 (SAS Institute, Cary, NC).

## Results

###  Baseline data

**Fig. 1:**
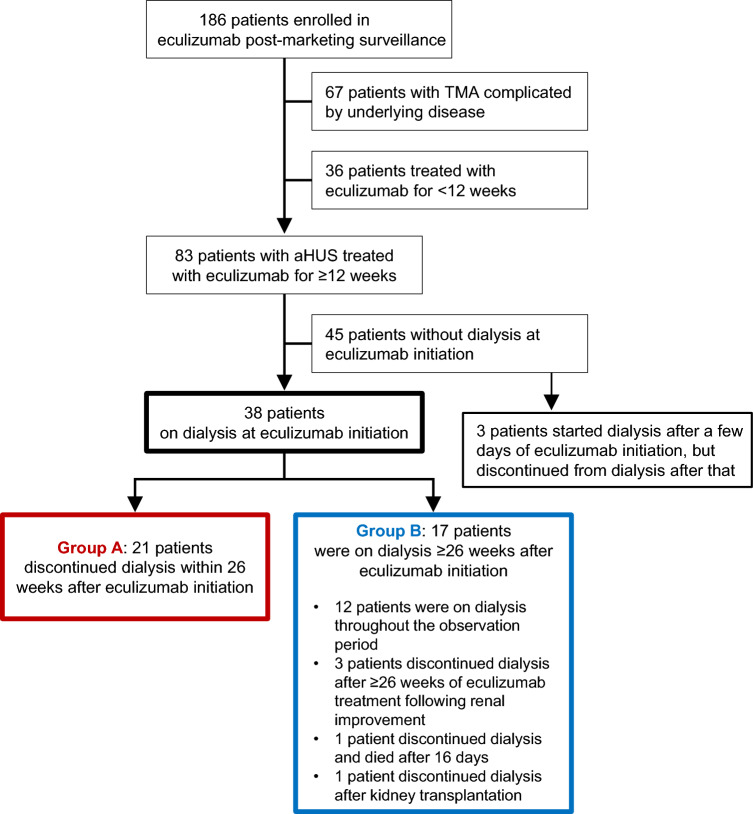
Patient selection flow and classification. A total of 38 patients with aHUS were on dialysis at eculizumab initiation in the post-marketing surveillance, of whom 21 were categorised in Group A and 17 were categorised in Group B. Group A: patients who discontinued dialysis within 26 weeks after eculizumab initiation. Group B: patients who were on dialysis ≥ 26 weeks after eculizumab initiation. Patients with haematopoietic stem cell transplantation TMA, malignancy-related TMA, drug-induced TMA and autoimmune disease TMA, were excluded. *aHUS* atypical haemolytic uraemic syndrome; *TMA* thrombotic microangiopathy

Of the 83 patients who were treated with eculizumab for ≥ 12 weeks, 38 (45.8%) were on dialysis at the start of eculizumab treatment. Of these 38 patients, 21 (55.3%) discontinued dialysis (Group A) and 17 (44.7%) did not discontinue dialysis (Group B) within 26 weeks of eculizumab treatment (Fig. 1). Among patients who were not on dialysis at the initiation of eculizumab, three started dialysis after a few days of eculizumab initiation, but discontinued dialysis during the observation period (Fig. 1, Online resource Table S1). These three patients were not included in any further analysis in this study.

### Patient characteristics and clinical course before eculizumab treatment

Baseline patient characteristics and clinical courses are summarised in Tables 1 and 2. There were no significant differences in age at first eculizumab administration, the duration of eculizumab treatment, or laboratory parameters (urine protein level, platelet count, LDH level, and haemoglobin level) between Groups A and B. The proportion of patients with complement-related gene variants was also similar between Groups A and B (9/19 [47.4%] vs. 6/13 [46.2%], *p* = 1.000) (Table 1). Additionally, there was no significant difference in the ratios of patients with each complement-related gene variant between Group A and Group B (Online resource Table S2). Hypertension as a complication was less frequent in Group A than in Group B (6/21 [28.6%] vs. 11/17 [64.7%], *p* = 0.022). The median (Q1–Q3) duration of dialysis before initiation of eculizumab (6 [4.0–10.0] vs. 17 [8.0–36.0] days, *p* = 0.011) and time from the TMA onset to the initiation of eculizumab (9 [7.0–18.0] vs. 25 [15.0–42.0] days, *p* = 0.008) were shorter in Group A than in Group B. In contrast, there was no significant difference in the median (Q1–Q3) time from TMA onset to initiation of plasma therapy between the two groups (3.5 [2.0–7.5] vs. 8.0 [3.0–10.0] days, *p* = 0.154).

**Table 1:** Demographic and clinical characteristics of patients at the initiation of eculizumab treatment

	Overall	Group A (dialysis discontinuation within 26 weeks)	Group B (no dialysis discontinuation within 26 weeks)	*p-*value
Total number of patients	38 (100.0)	21 (55.3)	17 (44.7)	–
Sex				
Male	17 (44.7)	9 (42.9)	8 (47.1)	1.000
Female	21 (55.3)	12 (57.1)	9 (52.9)	–
Child/Adult				
Child (< 18 years)	9 (23.7)	7 (33.3)	2 (11.8)	0.148
Adult (≥ 18 years)	29 (76.3)	14 (66.7)	15 (88.2)	–
Age (years)				
Child (< 18 years)	6 (3.0–16.0)	6 (3.0–16.0)	8 (0.0–16.0)	1.000
Adult (≥ 18 years)	58 (38.0–66.0)	54.5 (37.0–66.0)	62 (38.0–67.0)	0.484
History of kidney transplantation				
No	38 (100.0)	21 (100.0)	17 (100.0)	–
Yes	0 (0)	0 (0)	0 (0)	–
Diagnosis of hypertension				
No	21 (55.3)	15 (71.4)	6 (35.3)	0.022
Yes	17 (44.7)	6 (28.6)	11 (64.7)	–
Gene variant/anti-complement factor H antibody				
Number of patients	32	19	13	–
Not detected	13 (40.6)	8 (42.1)	5 (38.5)	1.000
Detected	15 (46.9)	9 (47.4)	6 (46.2)	–
Unknown	4 (12.5)	2 (10.5)	2 (15.4)	–
Clinical course before eculizumab				
Days of dialysis before the initiation of eculizumab treatment	8.5 (5.0–18.0)	6 (4.0–10.0)	17 (8.0–36.0)	0.011
Plasma therapy before administration of eculizumab				
No	2 (5.4)	2 (9.5)	0 (0.0)	0.492
Yes	36 (94.6)	19 (90.5)	17 (100.0)	–
Days of plasma therapy before administration of eculizumab	6 (3.0–11.5)	5 (3.0–8.0)	7 (4.0–15.0)	0.197
Days from TMA onset to initiation of plasma therapy	4 (2.0–9.0)	3.5 (2.0–7.5)	8 (3.0–10.0)	0.154
Days from TMA onset to the first administration of eculizumab	15 (7.0–31.0)	9 (7.0–18.0)	25 (15.0–42.0)	0.008
Laboratory values at the first dose of eculizumab				
Urine protein assay (mg/day)	300.00 (170.00–300.00), *n* = 5	300.00 (196.80–2,215.00), *n* = 4	170.00 (NA–NA) *n* = 1	0.716
Platelet count (× 10^9^/L)	63.5 (33.3–128.0)	66.0 (18.0–153.0)	61.0 (40.0–108.0)	0.628
Lactate dehydrogenase (IU/L)	528.5 (320.0–1,214.0)	487.0 (320.0–1,473.0)	570 (327.0–721.0)	0.445
Haemoglobin (g/dL)	8.10 (7.00–8.90)	7.40 (6.80–9.10)	8.20 (7.40–8.70)	0.757

Unless stated otherwise, data are *n* (%) or median (Q1–Q3); *p*-values were calculated using Fisher’s Exact test or Wilcoxon test

*NA* not applicable; *Q* quartile; *TMA* thrombotic microangiopathy

**Table 2:** Clinical course after initiation of eculizumab treatment and its outcomes

Item	Overall	Group A (dialysis discontinuation within 26 weeks)	Group B (no dialysis discontinuation within 26 weeks)	*p-*value
Number of patients	38 (100.0)	21 (55.3)	17 (44.7)	
Treatment period (weeks)	52.5 (30.0–115.0)	75.0 (32.0–133.0)	40.0 (26.0–82.0)	0.172
Observation period (weeks)	104 (37.0–158.0)	133 (81.0–155.0)	52 (34.0–158.0)	0.150
Outcome				
Survival	35 (92.1)	20 (95.2)	15 (88.2)	0.577
Death	3 (7.9)	1 (4.8)	2 (11.8)	–
Dialysis discontinuation after 26 weeks by the end of observation period	26 (68.4)	21 (100.0)	5 (29.4)	–
Time from initiation of eculizumab to discontinuation from dialysis (days)	17.5 (7.0–53.0)	12 (6.0–22.0)	442 (259.0–464.0)^a^	–

Unless stated otherwise, data are *n* (%) or median (Q1–Q3)

*Q* quartile

^a^The median (Q1–Q3) days from initiation of eculizumab treatment to dialysis discontinuation of five patients who discontinued after 27 weeks

### Dialysis discontinuation

During the observation period, 26 (68.4%) patients discontinued dialysis, of whom 21 (80.8%) in Group A discontinued within 26 weeks, and five (19.2%) in Group B discontinued dialysis after 27 weeks (Table 2). Overall, the median (Q1–Q3) time to dialysis discontinuation was 17.5 (7.0–53.0) days, 12 (6.0–22.0) days in Group A, and 442 (259.0–464.0) days in Group B (Table 2). No patient resumed dialysis after dialysis discontinuation. The estimated percentage of patients who remained on dialysis over the course of eculizumab treatment is shown in Online resource Fig. S1.

The background and clinical courses of the five patients in Group B who discontinued dialysis after 27 weeks of eculizumab treatment are summarised in Online resource Table S3 and Online resource Fig. S2. One adult patient (#1) died due to recurrence and deterioration of duodenal papilla cancer 16 days after dialysis discontinuation. A paediatric patient (#2) underwent kidney transplantation 184 weeks after eculizumab initiation and discontinued dialysis: eculizumab was continued thereafter. Three adult patients (#3, #4, and #5) discontinued after 63, 37, and 66 weeks of eculizumab treatment, respectively.

### Factors associated with dialysis discontinuation

Results from the unadjusted analysis suggested that hypertension as a complication (OR 4.583, 95% CI: 1.161–18.096, *p* = 0.029) and ≥ 15 days from TMA onset to the initiation of eculizumab administration (OR 11.665, 95% CI: 2.438–55.823, *p* = 0.002) reduced the number of patients discontinuing dialysis (Table 3). In adjusted analysis, the time from TMA onset to the initiation of eculizumab administration was independently associated with dialysis discontinuation: ≥ 15 days from TMA onset to the initiation of eculizumab administration tended to decrease the number of patients discontinuing dialysis (OR 10.068, 95% CI: 1.618–62.663, *p* = 0.013) (Table 3).

**Table 3:** Stepwise logistic regression analysis of dialysis discontinuation within 26 weeks

Background factor	Category	Unadjusted		Adjusted	
		OR (95% CI)	*P-*value	OR (95% CI)	*P-*value
Age at first dose, years	Adult or Child	3.750 (0.663–21.196)	0.134	0.832 (0.093–7.428)	0.869
Diagnosis of hypertension	Yes or No	4.583 (1.161–18.096)	0.029	3.134 (0.644–15.246)	0.157
Time from TMA onset to eculizumab initiation	≥ 15 or < 15 days	11.665 (2.438–55.823)	0.002	10.068 (1.618–62.663)	0.013

*CI* confidence interval; *OR* odds ratio; *TMA* thrombotic microangiopathy

### Changes in haematologic and renal parameters in patients with/without dialysis discontinuation within 26 weeks during eculizumab treatment

The ratio of patients who achieved haematologic normalisation (e.g., platelet counts and LDH levels) and kidney function improvement were estimated by Kaplan–Meier analysis (Online resource Fig. S3). Platelet count normalisation, LDH level normalisation, and kidney function improvement were achieved earlier in Group A than in Group B (*p* = 0.050, 0.014, and < 0.001, respectively). The Kaplan–Meier analysis also suggested that 75.0%, 37.5% and 41.2% of patients in Group B met the criteria of platelet count normalisation, LDH level normalisation, and kidney function improvement 26 weeks after eculizumab treatment initiation, respectively (Online resource Fig. S3).

### Correlations between the time from TMA onset to the initiation of eculizumab and clinical parameters

The relation between days from TMA onset to initiation of eculizumab treatment and the change of eGFR in each patient are shown in Online resource Fig. S4. The change in eGFR 26 weeks after initiation of eculizumab treatment was negatively correlated with days from TMA onset to initiation of eculizumab treatment in Group A (*r* =  − 0.741, *p* = 0.001), but no correlation was observed in Group B (*r* =  − 0.296, *p* = 0.325) (Online resource Fig. S4a-c). A similar relationship was observed in the platelet count changes 14 days after initiation of eculizumab treatment, which were negatively correlated with days from TMA onset to initiation of eculizumab treatment in Group A (*r* =  − 0.742, *p* < 0.001), but no correlation was observed in Group B (*r* =  − 0.029, *p* = 0.909) (Online resource Fig. S4d-f). Interestingly, the changes in platelet count 14 days after initiation of eculizumab treatment and in eGFR 26 weeks later were positively correlated in the total population (*r* = 0.801, *p* < 0.001), Group A (*r* = 0.720, *p* = 0.003), and Group B (*r* = 0.670, *p* = 0.012) (Online resource Fig. S4g-i).

## Discussion

Little is known about the patient characteristics and clinical courses related to dialysis discontinuation in patients with aHUS treated with a C5 inhibitor. This real-world study confirms that a short time (< 15 days) from TMA onset to initiation of eculizumab was positively associated with dialysis discontinuation in Japanese patients with aHUS.

Previous reports from clinical trials and real-world studies have demonstrated that earlier initiation of C5 inhibitor treatment following TMA onset contributed to better renal outcomes in patients with aHUS [12, 15, 16]. In a post hoc analysis of eculizumab pivotal studies, early eculizumab initiation led to eGFR improvement; mean eGFR improvement from baseline at 1 year in patients treated with eculizumab ≤ 7 days vs. > 7 days were 57 and 23 mL/min/1.73 m^2^, respectively (*p* = 0.0098) [15]. The analysis of adult patients in the post-marketing surveillance indicated that increasing time from the most recent TMA episode to eculizumab treatment was negatively associated with an improvement in serum creatinine levels [16]. Moreover, an analysis of the Japanese Diagnosis Procedure Combination database revealed that patients with eculizumab as first line treatment showed lower frequency and shorter duration of dialysis, and shorter duration and lower cost of hospitalization than those with first line plasma therapy [23]. Thus, the early initiation of C5 inhibitors as first line treatment would provide significant benefit, including renal recovery, in patients with aHUS.

The current study showed that 19.2% (5/26) of patients who discontinued dialysis did so 27 weeks after eculizumab initiation. A study of the long-term efficacy and safety of ravulizumab over 2 years also showed that the proportion of patients whose serum creatinine levels recovered ≥ 25% from baseline increased from 58.9% to 62.5% in adults and from 80.0% to 90.0% in paediatric patients after 26 weeks of treatment [24]. Expert opinion recommends the treatment with  C5 inhibitors for at least 6 to 12 months and 3 months after the normalization of kidney function, or for more than 12 months in patients who experienced severe extrarenal manifestations [25, 26].

In our study, platelet count change 14 days after eculizumab treatment was correlated with eGFR change 26 weeks after initiation of eculizumab treatment. Previous reports have suggested that platelet count and function could be affected by chronic kidney disease [27, 28]. Because the patients in Group B required long-term dialysis, they may have reached a state of chronic kidney dysfunction, which could have affected the time to improvement of platelet counts during eculizumab treatment. Although platelet count increase has been demonstrated to be an indicator for early response to C5 inhibitors [8, 29], relying solely on platelet counts during the early periods after treatment initiation may not be appropriate for assessing the response to C5 inhibitors in patients on dialysis.

A limitation of this study is the small sample size, which prevented robust analysis to investigate patient characteristics associated with dialysis discontinuation. The stage of chronic kidney disease before the initiation of eculizumab treatment, which can be correlated with dialysis discontinuation, was not collected in this post-marketing surveillance. Additionally, data on kidney function, such as proteinuria and urine volume, were not consistently collected, which could be an obstacle to understanding the renal improvement that may lead to dialysis discontinuation.

In conclusion, early initiation of eculizumab is a key factor for improved renal outcomes in patients with aHUS on dialysis. Because dialysis discontinuation can occur after long-term treatment and platelet improvement can take longer in some patients treated with C5 inhibitors, the effects of C5 inhibitors should be carefully and continuously evaluated.

## Supplementary Information

Below is the link to the electronic supplementary material.

Supplementary file1 (DOCX 386 kb)

## Data Availability

Alexion, AstraZeneca Rare Disease will consider requests for disclosure of clinical study participant-level data provided that participant privacy is assured through methods like data de-identification, pseudonymization, or anonymization (as required by applicable law), and if such disclosure was included in the relevant study informed consent form or similar documentation. Qualified academic investigators may request participant-level clinical data and supporting documents (statistical analysis plan and protocol) pertaining to Alexion-sponsored studies. Further details regarding data availability and instructions for requesting information are available in the Alexion Clinical Trials Disclosure and Transparency Policy at https://www.alexionclinicaltrialtransparency.com/data-requests/. Link to Data Request Form: https://alexion.com/contact-alexion/medical-information.
