# Supplementary material for: Characteristics and clinical courses of patients with atypical haemolytic uraemic syndrome on dialysis withdrawal after eculizumab treatment: sub-analysis of post-marketing surveillance in Japan
Source: J Nephrol. 2025 Oct 28;38(9):2663–71. doi: 10.1007/s40620-025-02433-z (PMC12712043; doi:10.1007/s40620-025-02433-z)
Supplement: Supplementary file 1 — Supplementary file1 (DOCX 386 kb) [file 40620_2025_2433_MOESM1_ESM.docx]

# **Online resources**

**Characteristics and clinical courses of patients with atypical haemolytic uraemic syndrome on dialysis withdrawal after eculizumab treatment: sub-analysis of post-marketing surveillance in Japan**

*Journal of Nephrology*

Shuichi Ito^1^, Masanori Matsumoto^2^, Akihiko Shimono^3^, Hirofumi Teranishi^3^, Shoichi Maruyama^4^

^1^Department of Pediatrics, Graduate School of Medicine, Yokohama City University, Kanagawa, Japan

^2^Department of Hematology and Blood Transfusion Medicine, Nara Medical University, Nara, Japan

^3^ Medical Affairs Division, Alexion Pharma GK, Tokyo, Japan

^4^Department of Nephrology, Nagoya University Graduate School of Medicine, Aichi, Japan

## Corresponding author:

Shuichi Ito

[itoshu@yokohama-cu.ac.jp](about:blank)

**Table S1** Patients who initiated dialysis after initiation of eculizumab treatment

| 0-year-old male. He started eculizumab after 47 days from the onset of TMA. Two days after starting eculizumab, the patient started dialysis and discontinued dialysis 69 days later.  At the time of initiating eculizumab treatment, the patient had oedema and proteinuria, complications of hypertension and liver dysfunction.  His platelet count, LDH level, and serum creatinine were 73 ×10^9^/L, 833 IU/L, and 0.9 mg/dL, respectively, at the initiation of eculizumab. Clinical data were not recorded at the time of dialysis discontinuation. |
| --- |
| Male in his 50s. He started eculizumab 24 days after the onset of TMA. He started dialysis the day after initiation of eculizumab and discontinued dialysis 5 days later.  At the time of initiation of eculizumab, the patient had hypertension, hyperuricaemia and insomnia.  His platelet count, LDH level, and serum creatinine were 40 ×10^9^/L, 755 IU/L, and 21.5 mg/dL, respectively, at the initiation of eculizumab, and 237 ×10^9^/L, 517 IU/L, and 5.9 mg/dL at the time of dialysis discontinuation. |
| Female in her 40s. She started eculizumab after 10 days from onset of TMA. She started dialysis the day after starting eculizumab and discontinued dialysis 50 days later.  At the time of starting eculizumab treatment, the patient had multiple sclerosis.  Her platelet count, LDH level, and serum creatinine were 62 ×10^9^/L, 892 IU/L, and 3.1 mg/dL, respectively, at the initiation of eculizumab; and 130 ×10^9^/L, 558 IU/L, and 3.69 mg/dL, respectively, at the time of dialysis discontinuation. |

Abbreviations: LDH = lactate dehydrogenase; TMA = thrombotic microangiopathy

**Table S2** Details of complement-related gene variants

|  | **Overall** | **Group A**  **(dialysis discontinuation within 26 weeks)** | **Group B**  **(no dialysis discontinuation within 26 weeks)** | ***p-*value** |
| --- | --- | --- | --- | --- |
| Gene variant tested, *N* | 32 | 19 | 13 |  |
| Gene variant detected, *n/N* (%) | 15/32 (46.9) | 9/19 (47.4) | 6/13 (46.2) | 1.000 |
| C3, *n/N* (%) | 6/32 (18.8) | 5/19 (26.3) | 1/13 (7.7) | 0.361 |
| CFH, *n/N* (%) | 8/32 (25.0) | 3/19 (15.8) | 5/13 (38.5) | 0.219 |
| CFB, *n/N* (%) | 2/32 (6.3) | 0 | 2/13 (15.4) | 0.157 |
| CFI, *n/N* (%) | 1/32 (3.1) | 0 | 1/13 (7.7) | 0.219 |
| MCP, *n/N* (%) | 1/32 (3.1) | 1/19 (5.3) | 0 | 0.401 |
| THBD, *n/N* (%) | 1/32 (3.1) | 0 | 1/13 (7.7) | 0.219 |

*p*-values were calculated using Fisher’s Exact test.

Abbreviations: CFB = complement factor B; CFH = complement factor H; CFI = complement factor I; MCP = membrane cofactor protein; THBD = thrombomodulin

**Table S3**  Details of patients who discontinued dialysis after 27 weeks from initiation of eculizumab treatment

|  | **Patient number** | | | | |
| --- | --- | --- | --- | --- | --- |
|  | **#1^a^** | **#2^b^** | **#3** | **#4** | **#5** |
| Sex | Male | Female | Male | Female | Male |
| Age, years | 70s | 0 | 50s | 60s | 20s |
| Comorbidity | Hypertension, malignant tumours | Pneumonia | Hypertension, gastritis, insomnia, increased inflammation | None | None |
| PE/PI before eculizumab, days | 57 | 1 | 15 | 6 | 13 |
| Dialysis before eculizumab, days | 58 | 17 | 17 | 8 | 22 |
| TMA onset to PE/PI, days | 4 | 2 | 16 | 4 | 9 |
| TMA onset to eculizumab, days | 61 | 18 | 33 | 15 | 31 |
| Duration of eculizumab treatment, days (weeks) | 185 (26) | 1,790 (256)  (continuing eculizumab) | 253 (36) | 155 (22) | 1,247 (178)  (continuing eculizumab) |
| Time from initiation of eculizumab treatment to dialysis discontinuation, days (weeks) | 190 (27) | 1,290 (184) | 442 (63) | 259 (37) | 464 (66) |
| Serum creatinine level (mg/dL) |  |  |  |  |  |
| At the first dose of eculizumab | 4.2 | 2.7 | 4.6 | 5.8 | 9.0 |
| At the discontinuation of dialysis | 2.9 | 0.6 | 2.8 | 3.4 | 2.5 |

^a^Died after 16 days from dialysis discontinuation due to recurrence and deterioration of duodenal papilla cancer

^b^Received renal transplantation after 1,290 days (184 weeks) after initiation of eculizumab treatment, and discontinued dialysis Eculizumab administration was continued thereafter.

Abbreviations: PE = plasma exchange; PI = plasma infusion; TMA = thrombotic microangiopathy

**
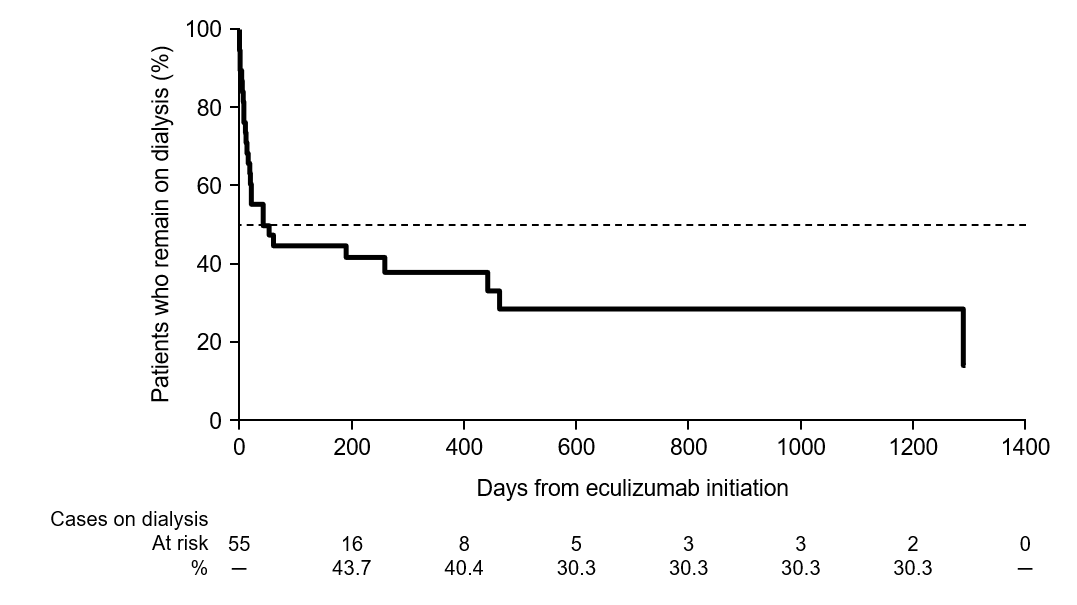
**

**Fig. S1** Time to dialysis discontinuation. The percentage of patients who remained on dialysis was estimated using the Kaplan–Meier analysis.

**Fig. S2** Haematologic and renal function parameter changes in patients who discontinued dialysis after 27 weeks from initiation of eculizumab treatment. (a–e) Five patients’ clinical parameters, including platelet count (blue circle), haemoglobin level (green triangle), serum creatinine level (purple rhombus), and LDH level (red square), at diagnosis of aHUS, eculizumab initiation, 14 days, 26 weeks, and before dialysis discontinuation were plotted.

Abbreviations: aHUS = atypical haemolytic uraemic syndrome; LDH = lactate dehydrogenase; Hb = haemoglobin; PLT = platelet count; sCr = serum creatinine

**
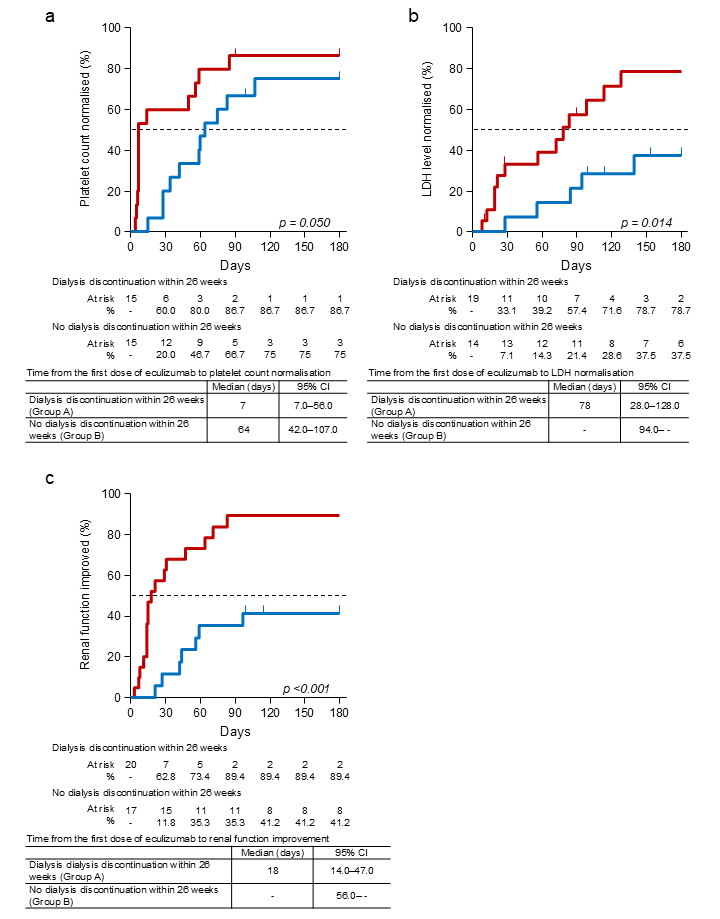
**

**Fig. S3** Time to achieve effectiveness endpoints. The percentages of participants achieving (a) platelet count normalisation, (b) LDH level normalisation, and (c) renal function improvement in Group A (patients who discontinued from dialysis within 26 weeks in red) and Group B (patients who stayed on dialysis within 26 weeks in blue) were estimated using Kaplan–Meier analysis, and were compared by using log-rank tests. The median days to achieve each endpoint and 95% CI were also estimated using Kaplan–Meier analysis.

Abbreviations: CI = confidence interval; LDH = lactate dehydrogenase

**Fig. S4** Relations between the change of parameters and days from TMA onset to eculizumab start. (a–c) Relations between the change in eGFR at 26 weeks after initiation of eculizumab treatment and days from TMA onset to initiation of eculizumab treatment, in the total population (black), Group A (patients who discontinued dialysis within 26 weeks in red), and Group B (patients who stayed on dialysis within 26 weeks in blue), respectively. (d–f) Relations between the change in platelet count at 14 days after initiation of eculizumab treatment and days from TMA onset to initiation of eculizumab treatment, in total population (black), Group A (red), and Group B (blue), respectively. (g–i) Relations between the change in eGFR and change in platelet count, in total population (black), Group A (red), and Group B (blue), respectively.

Abbreviations: eGFR = estimated glomerular filtration rate; TMA = thrombotic microangiopathy
